# ID 213 - Acesso aos Medicamentos Biológicos para Espondilite Anquilosante: uma análise do Ceaf em Minas Gerais (2018-2022)

**DOI:** 10.5327/2237-9622.2025.v34s1.213

**Published:** 2025-11-25

**Authors:** Bárbara Rodrigues Alvernaz dos Santos, Isadora Matheus Vilela de Lima, Francisco de Assis Acurcio†, Grazielle Dias Silva, Juliana Alvares Teodoro

**Keywords:** acesso, medicamentos biológicos, espondilite anquilosante, SUS

## Abstract

**Introdução:**

A Assistência Farmacêutica do SUS é dividida em três componentes. O Componente Especializado da Assistência Farmacêutica (Ceaf) é direcionado a doenças cujo tratamento é de média/alta complexidade e inclui tecnologias de alto custo, como os medicamentos modificadores do curso da doença (MMCD) biológicos. Estes constam no Protocolo Clínico e Diretriz Terapêutica da espondilite anquilosante (EA), uma doença reumática que pode evoluir com limitação funcional e acometimentos graves. Em Minas Gerais, os pacientes enfrentam morosidade no acesso aos MMCD biológicos por meio do Ceaf e, diante dessas questões, é necessário avaliar a execução do componente e buscar soluções que agilizem o acesso aos medicamentos. Assim, os objetivos deste estudo foram identificar o tempo de obtenção dos MMCD biológicos para tratamento da EA por meio do Ceaf em Minas Gerais, bem como analisar o tempo em cada uma das Unidades Regionais de Saúde (URS) e variáveis associadas a ele.

**Método:**

Por meio de um estudo transversal, foram coletadas informações clínicas, demográficas e perfil de uso de medicamentos de indivíduos com EA. Os dados foram coletados a partir de processos administrativos de solicitação de MMCD biológicos pelo SUS no estado de Minas Gerais, entre os anos de 2018 e 2022. O tempo para a obtenção de MMCD biológicos foi analisado a partir da data de abertura do processo até a dispensação do medicamento.

**Resultados:**

Dos 526 processos avaliados, foi identificado o tempo mediano de 53 dias (IIQ 33,00-82,75) para obtenção dos MMCD biológicos para EA. Uma análise detalhada revelou que a URS com o maior tempo mediano foi Manhumirim (mediana = 107,00; IIQ = 70,50-151,00). A regional com o menor tempo mediano foi Leopoldina (mediana = 27,50; IIQ = 18,75-36,75). As cinco regionais com menor tempo mediano de obtenção (Belo Horizonte, Itabira, Leopoldina, Ponte Nova e Ubá) representaram 27,3% dos processos. Já as cinco com maiores tempos medianos (Ituiutaba, Pirapora, Manhumirim, Uberaba e Varginha) corresponderam a 13,7% dos processos. Entre 2018 e 2022, observou-se uma redução no tempo médio dos processos ao longo dos anos. Em 2018, o tempo médio foi de 96 dias, caindo para 81 dias em 2019 e 2020, 58 dias em 2021, e atingindo 55 dias em 2022. De maneira similar, o tempo mediano também diminuiu, de 97 dias em 2018 para 52 dias em 2022, indicando uma tendência contínua de maior eficiência ao longo do período analisado.

**Conclusão:**

Apesar de observado o aumento na solicitação de medicamentos ao longo dos anos, foi demonstrada uma tendência de redução do tempo de obtenção durante o período. Um estudo anterior, também conduzido em Minas Gerais, entre o período de 2014 e 2016, revelou o tempo médio de 62,2 dias para dispensação de MMCD biológicos. Em relação a outros estados brasileiros, estudos sugeriram que o tempo médio de obtenção dos medicamentos do Ceaf, no estado de São Paulo e Santa Catarina, foi de 50 e 20 dias, respectivamente. Os dados deste trabalho sugerem uma otimização global do Ceaf-MG, possivelmente gerada pelos efeitos positivos das melhorias implementadas nos últimos anos, como a informatização das solicitações e a descentralização do Componente. Houve variabilidade entre as regionais, indicando possível distinção na qualificação dos serviços das URS. Espera-se que os resultados obtidos neste estudo possam se somar a pesquisas mais abrangentes, visando ao desenvolvimento de políticas e intervenções destinadas a otimizar o tempo de obtenção dos medicamentos do Ceaf em Minas Gerais.

